# Transcriptome Analysis of the Differentially Expressed Genes in the Male and Female Shrub Willows (*Salix suchowensis*)

**DOI:** 10.1371/journal.pone.0060181

**Published:** 2013-04-01

**Authors:** Jingjing Liu, Tongming Yin, Ning Ye, Yingnan Chen, Tingting Yin, Min Liu, Danial Hassani

**Affiliations:** Key Lab of Forest Genetics and Biotechnology, Nanjing Forestry University, Nanjing, China; Auburn University, United States of America

## Abstract

**Background:**

The dioecious system is relatively rare in plants. Shrub willow is an annual flowering dioecious woody plant, and possesses many characteristics that lend it as a great model for tracking the missing pieces of sex determination evolution. To gain a global view of the genes differentially expressed in the male and female shrub willows and to develop a database for further studies, we performed a large-scale transcriptome sequencing of flower buds which were separately collected from two types of sexes.

**Results:**

Totally, 1,201,931 high quality reads were obtained, with an average length of 389 bp and a total length of 467.96 Mb. The ESTs were assembled into 29,048 contigs, and 132,709 singletons. These unigenes were further functionally annotated by comparing their sequences to different proteins and functional domain databases and assigned with Gene Ontology (GO) terms. A biochemical pathway database containing 291 predicted pathways was also created based on the annotations of the unigenes. Digital expression analysis identified 806 differentially expressed genes between the male and female flower buds. And 33 of them located on the incipient sex chromosome of *Salicaceae*, among which, 12 genes might involve in plant sex determination empirically. These genes were worthy of special notification in future studies.

**Conclusions:**

In this study, a large number of EST sequences were generated from the flower buds of a male and a female shrub willow. We also reported the differentially expressed genes between the two sex-type flowers. This work provides valuable information and sequence resources for uncovering the sex determining genes and for future functional genomics analysis of *Salicaceae* spp.

## Introduction

Unlike animals, plants have no distinct germ line in the early developmental stage [1]. Instead, totipotent meristematic cells proceed through a long period of vegetative development before they eventually form complex sexual organs – the flowers [2]. Flowers are more varied than the equivalent structures of any other group of organisms [3]. For example, most of the cucumber plants are monoecious, but can be dioecious or hermaphroditic [4]–[5], whereas papaya is trioecious with all three sex types, male, female, and hermaphrodite [6]. The majority of plants are cosexuals [7], meaning individual plants have both sex functions, whether present within each flower (hermaphrodite), or in separate male and female flowers (monoecious). A minority of plant species are ‘sexually polymorphic’, including dioecious, gynodioecious, and androdioecious plants [8]. The dioecious system, with separate males and females, is of course the rule in most animals, but is found in only about 4% of angiosperm species [9].

The determinants of sexual phenotype in plants are diverse, ranging from sex chromosomes in *Marchantia polymorpha* and *Silene latifolia* to hormonal regulation in *Zea mays* and *Cucumis sativa*, and to pheromonal cross-talk between individuals in *Ceratopteris richardii* [4]. Sex chromosome systems have arisen several times in flowering plant evolution [8], and sex chromosomes are evolutionarily young in some plants while they are ancient in most mammals [10]–[11]. Thus, the sex determination evolution process which has done in mammals can be traced in plants.

*Genus Populus* and *genus Salix* are members of *Salicaceae*, a family of dioecious catkin-bearing woody plants [12]–[13]. Cytological studies reveal that most of the plant species in *Salicaceae* exist in the diploid form with a haploid number of chromosomes equal to 19 [12], [14], and there have been no definite sex chromosomes found in this family [15]–[16]. Yin et al. described the genetic and genomic features in the peritelomeric region of chromosome XIX that suggested this region in the *Populus* genome was in the process of developing characteristics of a sex chromosome [17]. However, the discovery of sex determining genes remains unresolved. Willows and poplars originate from the same ancestor [18]. Experimental studies show that their genomes share high colinearity [12], [13], [18]. *Salix suchowensis* is a native shrub willow species that distributes in the north of China, which can reach sexual maturity for reproduction in a year. Compare to poplar, *S. suchowensis* is a more desirable plant for discovering the sex determining gene(s) for *Salicaceae* spp.

Transcriptome sequencing has been proven to be an efficient way for gene discovery [19]–[22], especially with the availability of the high-throughput next generation sequencing technology. Many studies have been conducted for detecting the candidate genes underlying traits of interest in a variety of plant species, such as *Panicum hallii* [23], *Digitalis purpurea* [24], *Cajanus cajan* [25], *Bituminaria bituminosa* [26], *Pesea Americana* [27] and so on. In this study, we performed transcriptome sequencing for flower buds from the male and female trees of *S.suchowensis* using a 454 GS-FLX sequencer, thereby, to discover the differentially expressed genes in flower buds of two types of sexes. Base on homologus mapping in poplar genome, combining with various bioinformatics tools, we aim to generate a list of candidate genes that may involve in the sex determination of *Salicaceae* spp. This study will provide useful information for uncovering the sex determining genes and for reconstructing the regulatory network of sex determination for plants of *Salicaceae* in future.

## Results

### EST Sequence Generation and Assembly

We performed half a 454 GS FLX run on each of the two flower samples which bears only unique sexual type flower buds. Altogether, 1,201,931 reads were obtained, after quality control, 1,201,628 reads were left with an average length of 389 bp and a total length of 467.96 Mb, among which 629,683 were from female flower buds and 571,945 from male flower buds (Table 1).

**Table 1:** Statistics of willow ESTs generated by the 454 GS-FLX platform.

	Female	Male	Total
No. of reads	629,683	571,945	1,201,628
Average read length (bp)	375.86	404.09	389.30
Total bases (bp)	236,675,409	231,114,644	467,790,053
No. of reads in contigs	542,527	498,129	1,040,656
No. of reads as singletons	72,474	60,235	132,709

After primers and adaptors were removed, the ESTs generated in this project were subjected to cluster and assembly analysis. A total number of 161,757 unigenes were obtained,among which 29,048 were contigs and 132,709 were singletons. The contigs had an average length of 643 bp and a total length of approximately 20 Mb, while singletons only had an average length of 299 bp and a total length of approximately 40 Mb. There are 17,820 (61.35%) contigs greater than 400 bp, while only around 45,141 (34.03%) singletons are larger than 400 bp (Table 2). The size distribution of assembled contigs and singletons is presented in Figure S1.

**Table 2:** Statistics of willow unigenes.

	Contig	Singleton	Unigene
No. of sequences	29,048	132,709	161,757
Average read length (bp)	643.02	298.62	360.47
Total bases (bp)	18,677,872	39,630,050	58,307,922
No. of unigenes >400 bp	17,820	45,141	62,961
No. of unigenes aligned to *Populus* genome predicted genes	21,365	39,482	60,847
No. of unigenes aligned to nr databases	20,739	61,886	82,625

### Functional Annotation of Willow Transcriptome

Based on the alignments of willow unigenes to *Populus* genome predicted genes, a total of 60,846 annotative unigenes were obtained, among which 21,365 were contigs (73.55% of all the 29,048 contigs) and 39,481 were singletons (29.75% of the 132,709 singletons). These mappable unigenes covered 22,298 *Populus* genome predicted genes. There remained 7,683 contigs (26.45% of all the 29,048 contigs) and 93,228 singletons (70.25% of all singletons) unmappable to the *Populus* genome predicted genes. The annotative unigenes included 2,789 potential genes that corresponded to *Populus* genome predicted genes with unknown function.

To infer putative functions of the obtained unigenes, we further compared sequences of all unigenes against GenBank nonredundant protein database (nr). The analysis indicated that 20,739 contigs (71.39% of all the 29,048 contigs) and 61,886 singletons (46.63% of all the 132,709 singletons) had significant matches in the nr database (Table 2). Among the 20,739 contigs, 20,174 (∼97.28%) were *Populus* genome predicted genes and 565 were unmappable contigs. The unmappable contigs only accounted for a very low percentage of the 20,739 contigs (∼2.72%). We proposed that this might be due to the incomplete sequencing of poplar genome or correspond to the genes specific in willow genome. Among the 61,886 singletons that had significant matches in the nr database, only a small portion (8,484, ∼13.71%) were *Populus* genome predicted genes and the majority of them (53,402, ∼86.29%) were unmappable singletons.

From the above analyses, we can see that the majority of contigs are mappable and can be properly annotated both according to the *Populus* genome predicted genes and the nr database. Whereas to the singletons, a large portion (70.25%) are unmappable, and their annotation results are less reliable due to the relatively short sequences, most of which probably lack the conserved functional domains [5]. In this study, we focus on discovering genes differentially expressed between sexes. Singletons do not have enough numbers for statistical analysis [28]. Therefore, in the gene differential expression analysis, we only included the mappable singletons that were merged in the *Populus* genome predicted genes. The rest singletons were only used for function analysis.

**Figure 1:**
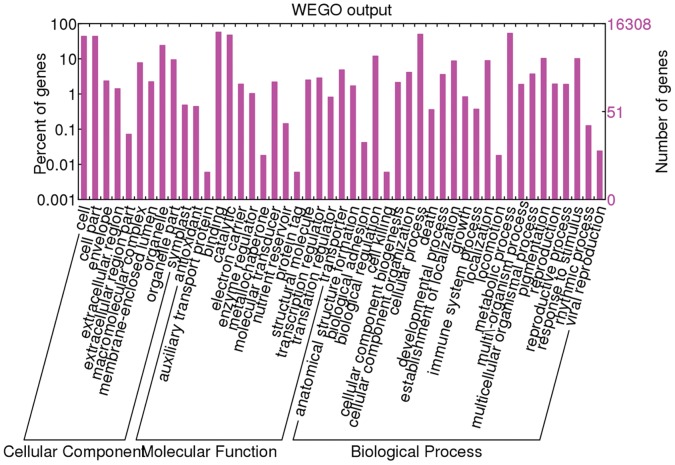
Number of willow unigenes in each functional category. Note: willow unigenes were classified into different functional groups based on a set of plant specific GO Slims within cellular component, molecular function and biological process categories.

After comparison with *Populus* genome predicted genes and nr database, the majority of the obtained unigenes were annotated, except for 4,543 contigs and 18,614 singletons (∼14.32% of all unigenes). Subsequently, Gene Ontology (GO) terms were further assigned to the obtained unigenes based on their sequence similarities to known proteins in the UniProt database annotated with GO terms as well as InterPro and Pfam domains they contained. A total of 16,308 unigenes were assigned at least one GO term. Among GO terms, 29,336 were in the biological process category, 20,248 in the molecular function category, and 22,586 in the cellular component category. These unigenes were further classified into different functional categories using a set of GO slims, which are a list of high-level GO terms providing a broad overview of the ontology content (http://www.geneontology.org/GO.slims.shtml). Figure 1 shows the functional classification of willow unigenes into plant specific GO slims within cellular component, molecular function and biological process categories. Among these categories, genes involving in cell, cell part, organelle, binding, catalytic, cellular process and metabolic process were the highest represented groups, indicating the flower buds were undergoing rapid growth and intensive metabolic activities. In biological process category, it was noteworthy that reproduction and reproductive process were highly presented, with 193 unigenes involved.

### Biochemical Pathways

We also identified the biochemical pathways of the willow ESTs generated in the present study to demonstrate their reliability for discovering the sex determining genes. Annotations of willow unigenes were fed into the KASS, and this process predicted a total of 291 pathways represented by a total of 6,820 unigenes. These predicted pathways represented the majority of plant biochemical pathways for biosynthesis of secondary metabolites, transcription and translation, energy metabolism and the progress of plant resistance and immunity.

We developed a database containing all the predicted willow pathways (Table S1). Enzymes catalyzing almost all steps in several major plant metabolic pathways including TCA cycle, oxidative phosphorylation, photosynthesis, glycolysis, gluconeogenesis, pentose phosphate pathway, and several important secondary metabolite biosynthesis pathways including carotenoid biosynthesis, flavonoid biosynthesis, cutin, suberine and wax biosynthesis, could be represented by unigenes derived from our EST collection. All these evidences demonstrated that the ESTs generated in this study provided a valuable resource for willow gene discovery and future functional analysis.

### Comparison of Transcriptomes between Dioecious Flower Buds

In analyzing the differentially expressed genes in the male and female flower buds, we included all of the assembled contigs (29,048) and those mappable singletons (39,482). By mapping them to the *Populus* genome predicted genes, these unigenes were further merged into a total of 29,981 genes, among which 22,298 were *Populus* genome predicted genes. The number reads of each gene were obtained using a custom PERL script. The digital expression profiling analysis identified 806 genes differentially expressed in the male and female flower buds with p≤0.0001 for all the employed statistics (Table S2), among which 417 genes showed significantly higher expression in male flower buds and 389 showed significantly higher expression in female flower buds. Since willow and poplar genome shared high colinearity [12], [13], [18], we mapped the differentially expressed genes onto the *Populus* scaffolds (http://www.phytozome.com/poplar.php, V2.0) using BLAT [29], allowing 80% sequence identity and 80% length coverage (Figure S2). Previous studies revealed that the gender locus were located on chromosome XIX in poplars [17], [30], the close relatives to willows. Chromosomal localization showed that there were 33 differentially expressed genes on chromosome XIX, with 17 highly expressed in female and 16 highly expressed in male (Table 3). These genes were of great interest for further investigation.

**Table 3:** Differentially expressed genes on chromosome XIX.

ID	Function distribution	high expressed
willow21195	NB-ARC domain-containing disease resistance protein	female
willow21197	Disease resistance protein (TIR-NBS-LRR class) family	male
willow21200	low-molecular-weight cysteine-rich 68	female
willow21210	Disease resistance protein (TIR-NBS-LRR class) family	female
willow21212	Disease resistance protein (TIR-NBS-LRR class)	female
willow21213	Disease resistance protein (TIR-NBS-LRR class) family	female
willow21226	flavonol synthase 1	male
willow21285	Disease resistance protein (TIR-NBS-LRR class) family	male
willow21343	NULL	male
willow21348	small and basic intrinsic protein 1A	male
willow21412	Plasma-membrane choline transporter family protein	male
willow21419	GroES-like zinc-binding dehydrogenase family protein	female
willow21446	Plant protein of unknown function (DUF827)	female
willow21463	Fasciclin-like arabinogalactan family protein	male
willow21521	Chalcone-flavanone isomerase family protein	female
willow21522	Chalcone-flavanone isomerase family protein	female
willow21527	Leucine-rich repeat protein kinase family protein	female
willow21541	Kinase interacting (KIP1-like) family protein	female
willow21568	Galactosyltransferase family protein	male
willow21569	Galactosyltransferase family protein	male
willow21604	Beta-glucosidase, GBA2 type family protein	male
willow21620	GDSL-like Lipase/Acylhydrolase superfamily protein	male
willow21626	K-box region and MADS-box transcription factor family protein	female
willow21693	laccase 14	female
willow21696	Plant protein of unknown function (DUF828)	male
willow21708	LOB domain-containing protein 2	male
willow21740	ribosomal protein L5 B	female
willow21752	expansin B3	female
willow21766	NULL	male
willow21772	Ankyrin repeat family protein	female
willow21774	Ankyrin repeat family protein	female
willow21790	Protein of unknown function (DUF789)	male
willow21809	NAD(P)-binding Rossmann-fold superfamily protein	male

A detail examination on the differentially expressed genes on chromosome XIX revealed that there was 1 MADS-box gene expressed significantly higher in female than in male. Since MADS-box genes are important in floral development [31], they are worthy of special notification. Previous genomic analysis revealed an overabundance of disease resistant genes in the vicinity region of gender locus in poplar genome [17]. The enrichment of disease resistant genes on chromosome XIX was supposed to play a significant role in triggering the divergence of the sex chromosome in *Populus* [17], [32]. In this study, we found 7 disease resistant genes on chromosome XIX which were differentially expressed in male and female, among which, 5 expressed significantly higher in female than in male and 2 expressed significantly higher in male than in female. Our results indicated that these genes were not merely presented in vicinity region of gender locus, but many of them were differentially expressed in two types of sexes. It was found that flavonoids play key roles in male fertility of some plants species [33], 3 genes of flavonoids pathway on chromosome XIX were found to express differentially, among which 2 expressed significantly higher in female and 1 expressed significantly higher in male rather than in female. It is also noteworthy that protein kinases were found to express differentially in different sex type flowers in cucumber [5]. We detected 2 protein kinases on chromosome XIX that were differentially expressed in the female and male flower buds of willow, among which 1 expressed significantly higher in male than in female and 1 expressed significantly higher in female than in male. Therefore, the aforementioned 12 genes are of special interest for the further exploring.

## Discussion

*Salix suchowensis* is a shrub willow. It has much smaller body size and relatively shorter juvenile period in comparison with that of many other tree species. Moreover, plant species in family of *Salicaceae* are dioecious and they possess an evolutionarily young sex chromosome [17]. All the above characteristics lend it as a great model for functional genomic studies of woody plants, and for exploring the missing pieces of sex determination evolution. Willow and poplar are closely related species. Comparison of *Populus* and *Salix* orthologous genes revealed that all modern *taxa* in the family of *Salicaceae* were descendents of a common progenitor whose genome underwent a whole-genome duplication event (known as “Salicoid duplication”) [18]. And alignment of the willow linkage map to the *Populus* genomic sequence revealed macrosynteny between willow and poplar genomes [12]. The complete genome sequencing of *Populus* [18] has shed light to genomic studies on willow species. However, sequence information of willow is scarce. In this study, a large ESTs collection (over 1.2 million reads) was obtained. These ESTs provide a valuable functional genomic resource to the *Salicaceae* research community.

Based on the alignments of willow unigenes to *Populus* genome predicted genes, about 73.55% of the obtained contigs were mappable, while only about 29.75% of the obtained singletons could be mapped to the *Populus* genome predicted genes. Annotation with the public databases, 74.84% contigs had significant matches. By contrast, only 46.63% of singletons could be annotated. Meanwhile, about 97.28% contigs that could be annotated using the public databases were mappable to the *Populus* genome predicted genes. This percentage decreased to 2.72% for the singletons. The inconsistency between contigs and singletons may largely due to the difference in their sequence lengths. The average length of contigs was 643 bp, while singletons only had an average length of 299 bp. In general, the longer the sequence the higher the chance of annotation and number of GO terms recovered [28]. Indeed, there was only a relatively small portion of contigs remained unannotated. The short unannotated contigs might correspond to 3′ or 5′ untranslated regions, non-coding RNAs, or sequences not containing known protein domains. As for the unannotated contigs longer than 500 bp, they had a high chance of corresponding to novel or undescribed genes. Since a large number of singletons remained unmapped and unannotated, we further examined the quality of these data. Results revealed that about 30.55% of them had no significant matches with plants sequences. Obviously, contigs are much more closely related to the *Populous* genome predicted genes than the singletons. Consider that the quality of singletons was less reliable, and singletons did not have enough numbers for statistical analysis [28], we only included the mappable singletons that were merged in the *Populus* genome predicted genes in the gene differential expression analysis.

Based on full length cDNA analysis, poplar genes have an average full length cDNA of 1,045 bp (from the beginning of the 5′UTR to the end of the polyA tail), ranging from 147 to 3,342 bp [34]. The average length of the obtained unigenes is much shorter than that of the full length cDNAs of poplar genes. Therefore, different contigs and singletons might be a part of the same gene. Based on the alignments of willow unigenes to *Populus* genome predicted genes, the mappable unigenes were merged into 29,981 genes. Thus the ESTs collection obtained in this study roughly covered about 66% of the total genes in willow genome; if total genes in willow were equivalent to that of *Populus* (45,033 predicted genes in *Populus* genome, http://www.phytozome.com/poplar.php, V2.0). Based on microarray analysis in *Arabidopsis*, about 55–67% genes expressed in a single tissue [35]. In human and mouse, around 60–70% genes expressed in a specific tissue [36]. Comparing to the results in the above organisms, the ESTs generated in this study were supposed to capture the majority of genes expressed in willow flower buds.

We performed read count analyses to identify differentially expressed ESTs based on sex. The identified number of differentially expressed genes was 806, which were over expressed either in the male or in the female. Since the individuals under analyzing were two different genotypes, even we specifically sequenced genes expressed in flower buds, the large number of differentially expressed genes were not only associated with gender differentiation. Thus those differentially expressed genes associated with flower development locating on the incipient sex chromosome are worthy of special notification in future analysis. However, merely based on differentially expression analysis, the exact sex determining gene(s) could not be identified. A feasible way is to examine the co-segregation of the differentially expressed genes with gender phenotype in a mapping pedigree. We have established a mapping pedigree with the sequenced male and female as parents, and we will conduct the co-segregation analysis when the phenotypic data are available.

If we know the location of sex determining genes in the genome, it will provide essential information to narrow down the candidate genes. It was reported that in poplar genome, the gender determination locus were mapped on chromosome XIX [17], [18], [30], [37], and our study revealed multiple differentially expressed genes on this chromosome. Several kinds of evidence suggested the involvement of more than one locus in sex determination in plants [38], [39], [40], [41]. In close relatives of *S. suchowensis*, gender locus was mapped to different positions on the alternate chromatids in *Populus nigra*, suggesting there was more than one genetic locus involved in sex determination [30], and the observed sex ratios in certain genetic backgrounds of *Salix viminalis* also suggested a multilocus epistatic model of gender determination [42]. A genetic model of the evolutionary transition from cosexuality to dioecy suggested the evolution of two sexes must generally require at least two linked genetic loci [8], [43]. However, direct evidence supporting this hypothesis was scarce due to the unsuccessful cloning of the gender determining gene(s) in plants. The exact sex determining genes were not identified either in this study, but essential archive for future study was provided.

Including our study, extensive efforts have been exerted to detect the sex-specific expressed genes from developing flower buds or reproductive organs [5], [44], [45], yet have not led to discovery of the exact sex determining genes. It is noteworthy that sex-determination happens very early in flower development [39], so the differentially expressed genes identified are controlled in response to sex, rather than the controlling loci [8]. We conducted differential expression analysis with the unflushed flower buds in this study, hopefully to cover the controlling loci in the differentially expressed genes. Although some differentially expressed genes were found on the incipient sex chromosome, we can not establish the direct relationship of these genes with gender merely based on the current data. Moreover, genes known to be important in floral development appear not to have direct roles in sex determination [46], [47], thus study over a pool of putative regulatory elements for future functional analysis could also be essential. Nevertheless, our study provided some novel insights into the molecular mechanisms of willow sex determination, as well as a valuable functional genomics resource and a list of candidate genes functional analysis in future.

### Conclusion

In this study, we generated a large ESTs collection and identified a list of candidate genes that differentially expressed in the male and female flower buds of *S. Suchowensis*. It is noteworthy that we detected 33 differentially expressed genes located on the incipient sex chromosome, and 12 of them were of special interest for further investigation for their roles in sex determination. These data will be of considerable interest to the *Salicaceae* research community. Our study also provides an archive for future studies of the molecular mechanism underlying the evolutionary process from monoecy to dioecy, and can also be useful for future *de novo* sequencing of this shrub willow.

## Materials and Methods

### Flower Buds Collection and RNA Extraction

Male and female willows used in this study were planted by cuttings on campus of Nanjing Forestry University. Cuttings were sampled from Xinyi, Jiangsu province of China in 2009. Flower buds were collected separately from the male and female trees in Feb, 2011. We selectively collected the expanded but unflushed flower buds, removed the bud bracts, and froze the collected buds in liquid nitrogen for total RNA isolation. The field studies did not involve any endangered or protected species, and sample collection was authorized by the local government.

Total RNA was isolated using CTAB-LiCl method [48]. The integrity of total RNA was determined by gel electrophoresis, and RNA concentration was measured using a Nanodrop-2000 spectrophotometer (Thermo, Inc.). Then, DNA residues were digested by DNase (TaKaRa, Inc.) at 37°C for 30 minutes. Subsequently, the extracted RNA was purified using Oligotex mRNA Mini Kit (Qiagen, Inc.). At last, size and concentration of mRNA were quantified using a RNA 6000 Pico Chip on the Agilent 2100 Bioanalyzer (Agilent, Inc.).

### Sequencing Library Construction and 454 Sequencing

cDNA synthesis was performed using cDNA Synthesis System Kit (Roche, Inc.). Sequencing libraries for the male and female flowers were separately constructed using Rapid Library Prep Kit (Roche, Inc.) following the manufacture’s protocol. Quality of the sequencing libraries were checked using the Agilent 2100 Bioanalyzer (Agilent, Inc.). Approximate 2.1 million beads for each of the two sex-type flowers were separately loaded into two sections of a pico-titer plate. Sequencing run was performed on a Roche 454 GS FLX sequencer at Nanjing Forestry University following the manufacturer’s protocols. All the ESTs were published in EMBL Nucleotide Archive (ENA) with an ArrayExpress accession number of E-MTAB-1445.

### Sequence Processing and Assembly

All the raw sequences were processed to remove low quality reads and adaptor sequences using programs LUCY [49] and SeqClean (http://compbio.dfci.harvard.edu/tgi/software/). Transcriptome sequences of the male and female flowers were subjected to *de novo* assembly using the 454 Newbler V2.7. We parsed the 454 ReadStatus.txt file to determine the singleton reads, which were not assembled with any other reads. Then the contig and singleton files were used to generate a unigene file.

### Gene Annotation and Pathway Prediction

All unigenes were submitted for homology and annotation searches, Gene Ontology (GO) annotation [50], and pathway analysis. The annotation of unigenes was performed by BLASTX [51] against the poplar protein database (http://www.phytozome.com/poplar.php, V2.0), NCBI non-redundant protein database (nr) and UniProt databases with a cutoff e value of 1e^−5^. The GO terms were assigned to each unigene based on the GO terms annotated to its corresponding homologues in the UniProt database [52], as well as those to InterPro and pfam domains using interpro2go and pfam2go mapping files provided by the GO website (http://www.geneontology.org), respectively. The annotation results were further plotted by WEGO, a web tool for plotting GO annotations (http://wego.genomics.org.cn/cgi-bin/wego/index.pl) [53]. Detailed annotation was then used to retrieve keywords to identify genes related to sex determining and flowering.

The metabolic pathway mapping was accomplished with KEGG Automatic Annotation Server (KAAS) (http://www.genome.jp/tools/kaas/) [54]. KAAS provides functional annotation of genes in a genome by BLAST comparisons against a manually curated set of ortholog groups in the KEGG GENES database. KAAS assigned each willow gene a KEGG Orthology (KO) number and these were subsequently mapped to one of KEGG’S reference metabolic pathways.

### Identification of Differentially Expressed Genes in Male and Female Flower Buds

Following cDNA sequence assembly and unigene mapping to poplar genome, transcript count information for sequences corresponding to each gene was associated with each of the corresponding flower tissue to obtain relative expression levels following normalization to the total number of sequenced transcripts per sample. Significance of differential gene expression was determined by using the R [55], χ^2^ and Fisher exact test statistics integrated in a freely available IDEG6 web tool (http://telethon.bio.unipd.it/bioinfo/IDEG6/) [56]. A gene was considered to be differentially expressed when all the above statistical tests yielded significance values ≤0.0001.

## Supporting Information

Figure S1 **Length distributions of willow unigenes.**
(DOC)

Figure S2 **Location of the differentially expressed genes on homologous chromosomes of** ***Populus.***
(JPG)

Table S1 **Biochemical pathways represented by the EST collection of this study.**
(XLS)

Table S2 **Genes differentially expressed in the male and female flower buds detected in this study.**
(XLS)
