# Supplementary figures and images for: Transcriptome Analysis of the Differentially Expressed Genes in the Male and Female Shrub Willows (Salix suchowensis)
Source: PLoS One. 2013 Apr 1;8(4):e60181. doi: 10.1371/journal.pone.0060181 (PMC3613397; doi:10.1371/journal.pone.0060181)

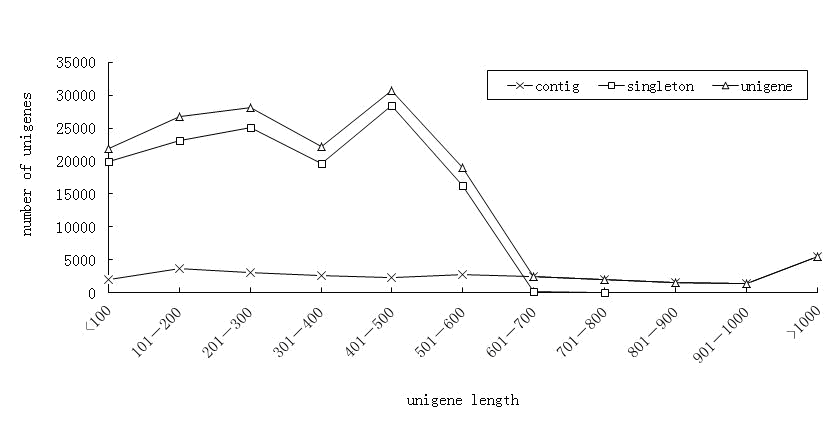

Supplement: Figure S1 — Length distributions of willow unigenes. (DOC) [file pone.0060181.s001.doc]

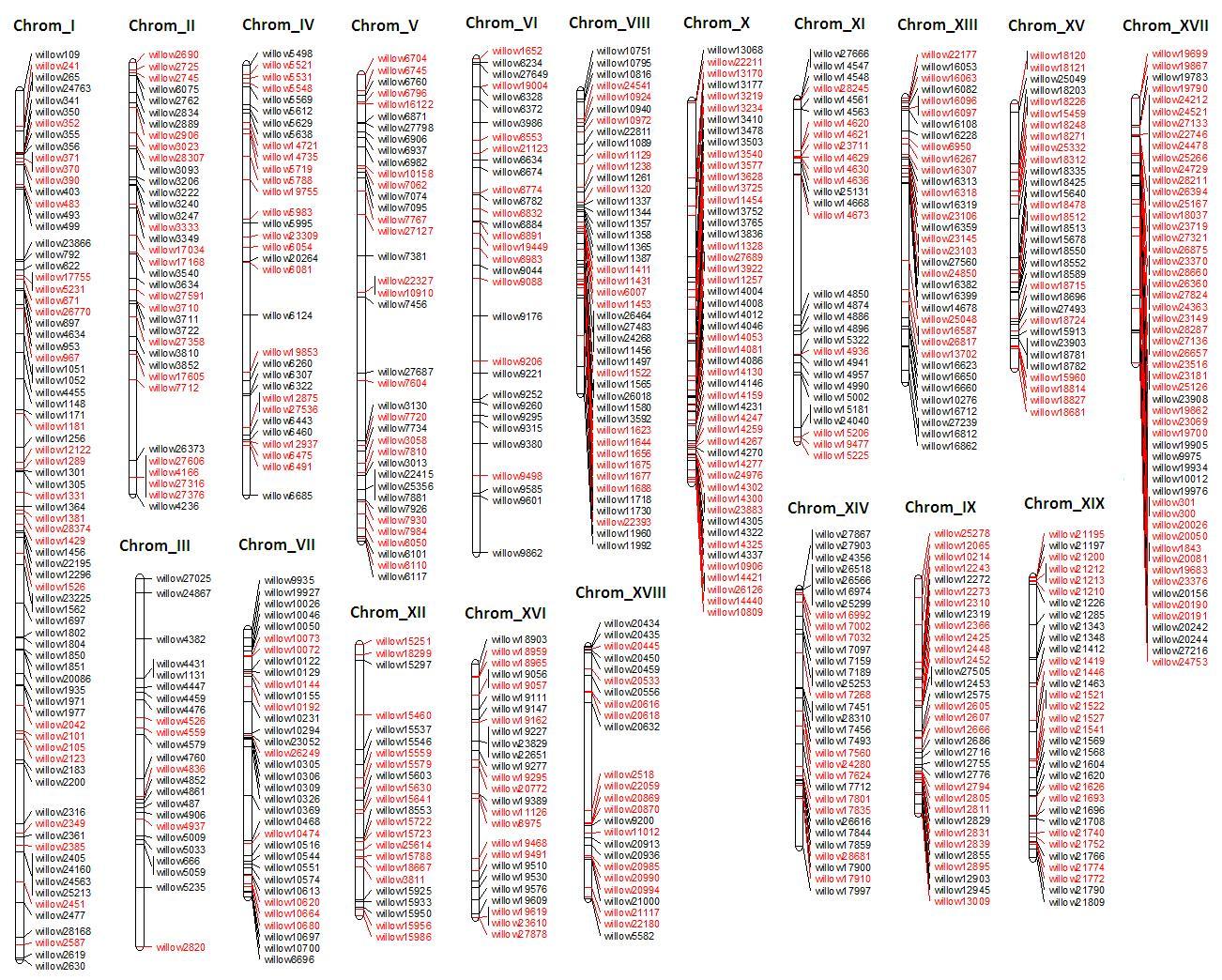

Supplement: Figure S2 — Location of the differentially expressed genes on homologous chromosomes of Populus. (JPG) [file pone.0060181.s002.jpg]
